# Neuroinflammation is increased in the parietal cortex of atypical Alzheimer’s disease

**DOI:** 10.1186/s12974-018-1180-y

**Published:** 2018-05-29

**Authors:** Baayla D. C. Boon, Jeroen J. M. Hoozemans, Boaz Lopuhaä, Kristel N. Eigenhuis, Philip Scheltens, Wouter Kamphorst, Annemieke J. M. Rozemuller, Femke H. Bouwman

**Affiliations:** 1 Department of Neurology, Alzheimer Center, Amsterdam Neuroscience, VU University Medical Center, Amsterdam, The Netherlands; 2 Department of Pathology, Amsterdam Neuroscience, VU University Medical Center, Amsterdam, The Netherlands

**Keywords:** Atypical Alzheimer’s disease, Microglia, Complement, Human brain tissue, Neuroinflammation, Amyloid-beta plaque

## Abstract

**Background:**

While most patients with Alzheimer’s disease (AD) present with memory complaints, 30% of patients with early disease onset present with non-amnestic symptoms. This atypical presentation is thought to be caused by a different spreading of neurofibrillary tangles (NFT) than originally proposed by Braak and Braak. Recent studies suggest a prominent role for neuroinflammation in the spreading of tau pathology.

**Methods:**

We aimed to explore whether an atypical spreading of pathology in AD is associated with an atypical distribution of neuroinflammation. Typical and atypical AD cases were selected based on both NFT distribution and amnestic or non-amnestic clinical presentation. Immunohistochemistry was performed on the temporal pole and superior parietal lobe of 10 typical and 9 atypical AD cases. The presence of amyloid-beta (N-terminal; IC16), pTau (AT8), reactive astrocytes (GFAP), microglia (Iba1, CD68, and HLA-DP/DQ/DR), and complement factors (C1q, C3d, C4b, and C5b-9) was quantified by image analysis. Differences in lobar distribution patterns of immunoreactivity were statistically assessed using a linear mixed model.

**Results:**

We found a temporal dominant distribution for amyloid-beta, GFAP, and Iba1 in both typical and atypical AD. Distribution of pTau, CD68, HLA-DP/DQ/DR, C3d, and C4b differed between AD variants. Typical AD cases showed a temporal dominant distribution of these markers, whereas atypical AD cases showed a parietal dominant distribution. Interestingly, when quantifying for the number of amyloid-beta plaques instead of stained surface area, atypical AD cases differed in distribution pattern from typical AD cases. Remarkably, plaque morphology and localization of neuroinflammation within the plaques was different between the two phenotypes.

**Conclusions:**

Our data show a different localization of neuroinflammatory markers and amyloid-beta plaques between AD phenotypes. In addition, these markers reflect the atypical distribution of tau pathology in atypical AD, suggesting that neuroinflammation might be a crucial link between amyloid-beta deposits, tau pathology, and clinical symptoms.

## Background

Patients with Alzheimer’s disease (AD) typically present with episodic memory impairment followed by deterioration of executive functioning, praxis, and visuospatial skills. However, AD patients may also present with an atypical phenotype [1, 2]. An atypical presentation is seen in 10% of the late-onset AD (LOAD) patients (≥ 65 years of age) and up to 30% of the early onset (< 65 years) AD (EOAD) patients [3]. So far, three variants of atypical AD have been described: the posterior cortical atrophy (PCA) variant characterized by visuoperceptual problems [4], the logopenic variant characterized by aphasia [5], and the frontal variant associated with behavioral changes [1, 2]. In addition to clinical differences, these different AD variants show syndrome-specific atrophy patterns on MRI [6].

AD is characterized by the deposition of amyloid-beta plaques and the formation of neurofibrillary tangles (NFT) in the brain. During disease progression, both plaques and NFTs are assumed to spread through the brain in a fixed order [7, 8]. However, the typical NFT distribution as originally described by Braak and Braak [9] does not seem to hold for all AD cases. Clinicopathological studies indicated that AD patients with an atypical phenotype have an atypical NFT distribution [9, 10]. Furthermore, this atypical NFT distribution was demonstrated in living AD patients using the tau tracer [^18^F]AV1451 [11]. While the atypical distribution of NFTs corresponds with the observed clinical phenotype, the cause of this difference in NFT spreading between AD variants remains elusive.

There is accumulating evidence that inflammation plays a prominent role in the pathogenesis of AD. Recently, genome-wide association studies have identified several genes involved in inflammation, especially those engaged in microglia function, as risk factors for developing AD [12–15]. The AD brain shows an increased presence of activated microglia, reactive astrocytes, proinflammatory cytokines, acute phase proteins, and activated complement proteins compared to controls [16]. Complement proteins co-localize with NFTs [17, 18], as well as with amyloid-beta deposits [19], and are actively involved in the formation of these pathological structures. Clusters of activated microglia are found in amyloid plaques, and the presence of activated microglia increases with disease severity [20, 21]. Recent disease models suggest that microglia are actively involved in the spreading of phosphorylated Tau (pTau) [22–24]. Tau pathology is heavily reduced in disease-modeled mice that are depleted for microglia compared to their microglia-positive peers [23, 24]. In the human brain, the presence of activated microglia correlates with Braak staging for NFTs [25].

Evidence for the correlation of pTau, neuroinflammation, and microglia in AD subtypes is lacking. In this study, we aimed to explore whether an atypical spreading of NFT pathology in non-amnestic AD is associated with an atypical distribution of neuroinflammation. In a well-defined cohort of typical and atypical AD, we assessed and compared the presence of pTau, amyloid-beta, (activated) glial cells, and complement proteins in temporal and parietal cortical areas.

## Methods

### Post-mortem brain tissue

Post-mortem brain tissue was obtained from the Netherlands Brain Bank (NBB; Amsterdam, The Netherlands). Donors signed informed consent for brain autopsy, and the use of brain tissue and medical records for research purposes. Neuropathological diagnosis was based on histochemical stainings including hematoxylin and eosin, congo red staining, Bodian or Gallyas and methenamine silver stainings, and immunohistochemical stainings for amyloid-beta, pTau, alpha-synuclein, and p62. These stainings were performed on formalin-fixed paraffin-embedded (FFPE) brain tissue of multiple brain regions including the frontal cortex, temporal pole, superior parietal lobe, occipital pole, amygdala, and the hippocampus. Neuropathological diagnosis of AD was based on Braak stages for NFT and amyloid [7], Thal phases for amyloid-beta [8], and CERAD criteria for neuritic plaques [26].

### Selection of typical and atypical AD cases

**Fig. 1:**
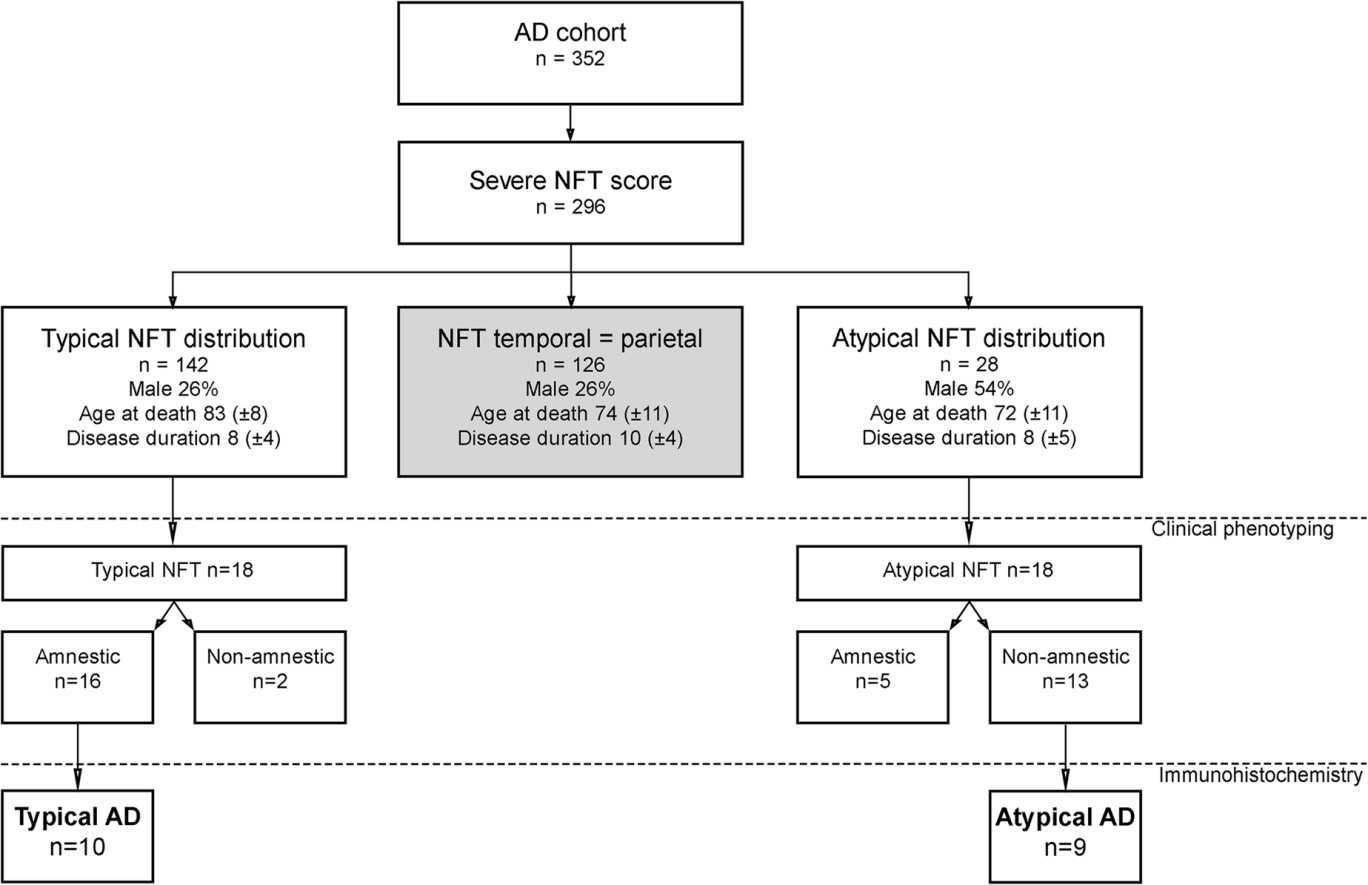
Flowchart of neuropathologically assessed and semi-quantitatively scored AD cohort. Between 1996 and 2014, 352 AD cases came to autopsy and were semi-quantitatively scored for NFTs as described by Hoogendijk et al. [27]. In 296 cases, an NFT score of ≥ 3 in the temporal and/or parietal cortex was observed. Typical NFT distribution was defined as a higher NFT score in the temporal compared to the parietal cortex. Atypical NFT distribution was defined as a higher NFT score in the parietal compared to the temporal section. From the cases with typical and atypical NFT distribution, 18 cases per group were selected for which the clinical phenotype was stratified as either amnestic or non-amnestic (results shown in Table 2). From these clinical phenotyped cases, 9 cases with an atypical NFT as well as non-amnestic clinical presentation were compared with 10 cases with an amnestic presentation and typical NFT distribution using immunohistochemistry. Mean ± SD is shown for age at death and disease duration in years. AD Alzheimer’s disease, NFT neurofibrillary tangle

Between 1996 and 2014, 352 AD cases came to autopsy and were semi-quantitatively scored by two neuropathologists (WK, AR) for the NFT load using Bodian or Gallyas staining in the temporal pole, the frontal, superior parietal, and occipital cortex as previously described by Hoogendijk et al. [27]. The NFT load was scored in a 0.4-mm^2^ area as being absent (0), sparse (1), mild (2; 2 to 3 NFTs), or severe (3; > 3 NFTs) for each brain region separately. From this cohort, we selected cases with an NFT score of 3 in either the temporal or parietal section, or in both sections, resulting in 296 cases (for flowchart, see Fig. 1). For 142 cases, the NFT score was higher in the temporal section than the parietal section. These cases were referred to as having a typical NFT distribution [7]. In 126 of 296 cases, an NFT score of 3 was found in the temporal as well as the parietal section. A higher NFT score in the parietal compared to the temporal section was observed in 28 cases and was defined as a parietal dominant and thus atypical NFT distribution.

To study the distribution of neuroinflammation in typical and atypical AD, we further refined our cohort to include only cases with a concordance between clinical presentation and NFT distribution. The clinical phenotype of 36 cases with atypical and typical NFT pathology was retrospectively assessed. In 18 out of 28 cases with an atypical NFT distribution, the available clinical information was sufficient to come to a retrospective clinical diagnosis (see Table 1 for demographics). To have an equal group for comparison, we randomly chose 18 cases with both typical NFT distribution and sufficient clinical information for clinical phenotyping. Clinical assessment was performed retrospectively and independently by 2 cognitive neurologists (YP and FB) using the NIA-AA criteria [2]. Both clinicians were blinded to the pathological stratification when assessing the clinical phenotype. The clinical stratification was based on (collateral) history and cognitive examination documented by the clinical neurologist. Cases with first-degree relatives affected by EOAD were excluded to minimize the risk of genetic AD. Other exclusion criteria were sepsis, other neurodegenerative or psychiatric diseases, significant cerebrovascular disease, post-mortem interval > 12 h, and prior known genetic mutations. Due to our exclusion criteria and availability of archived brain tissue samples, our inclusion was limited to 9 atypical AD cases and 10 typical AD cases of which the temporal pole and superior parietal lobe were assessed by immunohistochemistry (see Fig. 1 for inclusion flowchart, Table 2 for patient details, and Table 4 for demographics in the “Results” section). Cases were not intentionally matched for disease duration, brain weight, ApoE status, or post-mortem interval.

**Table 1:** Demographics of 36 cases with typical and atypical NFT distribution for which extensive retrospective clinical assessment was performed

	Typical NFT distribution *n* = 18		Atypical NFT distribution *n* = 18	
Phenotype	Amnestic (*n* = 16)	Non-amnestic (*n* = 2)	Amnestic (*n* = 5)	Non-amnestic (*n* = 13)
Male, *n* (%)	6 (37)	0	3 (60)	8 (62)
Age at death	82 (± 7)	88 (± 5)	71 (± 11)	67(± 7)
Disease duration	8 (± 5)	7 (± 4)	11 (± 5)	8 (± 4)
NFT stage [7]				
*n* per stage 4/5/6	2/10/4	1/1/0	0/2/3	0/7/6
Amyloid stage [7]				
*n* per stage O/A/B/C	0/0/16	0/0/2	0/0/5	0/1/12

Data are mean ± SD. Age at death and disease duration shown in years

*NFT* neurofibrillary tangle

**Table 2:** Clinical and neuropathological characteristics of typical and atypical AD cases

Case	Phenotype	Symptoms at clinical presentation	Sex	Age at death	Disease duration	NFT stage [7]	Amyloid stage [7]	Brain weight (grams)	Cause of death	PMI	ApoE
1	Typical AD	Memory	F	92	8	5	C	933	Heart failure	7:00	43
2	Typical AD	Memory, disorientation	F	84	4	4	C	908	Cardiogenic shock	4:15	32
3	Typical AD	Memory, disorientation	F	84	9	5	C	827	Cachexia	6:40	43
4	Typical AD	Memory	F	89	5	5	C	962	Pneumonia	6:28	43
5	Typical AD	Memory	F	83	6	6	C	1100	Dehydration	6:17	43
6	Typical AD	Memory, behavior	F	91	3	4	C	1026	Cachexia	6:25	33
7	Typical AD	Memory,	F	77	2	5	C	999	Pneumonia	6:05	33
8	Typical AD	Memory, behavior	M	70	2	6	C	1261	Metastasized colon carcinoma	6:20	43
9	Typical AD	Memory	F	76	12	5	C	1223	Unknown	10:45	44
10	Typical AD	Memory	M	60	2	6	C	1191	Cachexia	6:15	43
11	Atypical AD	Aphasia, dyscalculia, agraphia, left-right agnosia, visuoconstruction problems	F	65	6	6	C	975	Pneumonia	5:40	33
12	Atypical AD	Aphasia, dyslexia, apraxia, visuoconstruction problems	M	65	2	6	C	1057	Cardiac insufficiency	8:50	44
13	Atypical AD	Aphasia, acalculia, fingeragnosia, apraxia	M	64	7	5	C	1135	Pneumonia	4:45	42
14	Atypical AD	Parkinsonism, falling, alien hand syndrome	F	67	3	5	C	817	Epileptic insult	7:35	33
15	Atypical AD	Aphasia, apathy, agitation	M	59	6	6	C	1300	Cachexia	5:05	44
16	Atypical AD	Aphasia, dyscalculia, dyslexia, disorientation	M	62	3	6	C	1116	Malign neuroleptic syndrome	4:15	43
17	Atypical AD	Aphasia, dyslexia, apathy, apraxia	M	65	1	5	C	1150	Euthanasia	6:50	43
18	Atypical AD	Aphasia, dyslexia, apraxia, visuospatial problems, behavior	M	62	6	5	B	1153	Cachexia	4:40	33
19	Atypical AD	Visual hallucinations, psychosis	M	61	6	6	C	1355	Pneumonia	5:00	43

Age at death and disease duration in years; post-mortem interval in hours to minutes. Typical AD defined as more neurofibrillary tangles in the temporal compared to the parietal cortex by semi-quantitative scoring as described by Hoogendijk et al. [27] and an amnestic presentation during life. Atypical AD defined as more neurofibrillary tangles assessed by semi-quantitative scoring in the parietal compared to the temporal cortex and a non-amnestic presentation

*AD* Alzheimer’s disease, *F* female, *M* male, *NFT* neurofibrillary tangle, *PMI* post-mortem interval

### Immunohistochemistry (IHC)

IHC was performed to detect pTau (AT8); amyloid-beta (N-terminal; IC16); reactive astrocytes (GFAP); microglia (Iba1); activated microglia (CD68 and HLA-DP/DQ/DR); and complement proteins C1q, C3d, C4b, and C5b-9 (Table 3). FFPE sections (5-μm thick) from the temporal pole and superior parietal lobe of the right hemisphere were used.

**Table 3:** Characteristics of primary antibodies and staining details

Antibody	Antigen	Species	Origin details	Dilution	Incubation time	Antigen retrieval	Detection method
pTau, clone AT8	Tau phosphorylated at Ser202 and Thr205	Mouse IgG1	ThermoFisher, Pittsburgh, USA	1:10000	32 min at 36 °C	Heat-induced (pH 8.5) for 24 min	Optiview
Amyloid-beta, clone IC-16	N-terminus of amyloid-beta (aa 1-16)	Mouse IgG2a	Dr. Carsten Korth, University of Dusseldorf, Germany	1:25	32 min at 36 °C	Heat-induced (pH 8.5) for 24 min	Optiview
GFAP, clone EP672Y	Glial fibrillary acidic protein	Mouse	Roche, Basel, Switzerland	1: 2	8 min at 37 °C	Heat-induced (pH 8.5) for 32 min	Optiview
Iba1	C-terminus of Iba1	Rabbit	Wako Pure Chemical Industries, Osaka, Japan	1:4000	32 min at 36 °C	Heat-induced (pH 8.5) for 16 min	Optiview
CD68, clone KP1	CD68	Mouse IgG1	Dako, Glostrup, Denmark	1:1200	Overnight at 4°C	Heat-induced (pH 6.0) by autoclave	EnVision
HLA-DP/DQ/DR, clone CR3/43	Alpha and beta-chains of all products of the DP, DQ, and DR subregions	Mouse IgG1	Dako	1:800	Overnight at 4°C	Heat-induced (pH 6.0) by autoclave	EnVision
C1q	C1q	Rabbit	Dako	1:25600	Overnight at 4°C	Heat-induced (pH 6.0) by autoclave	EnVision
C3d	C3d	Rabbit	Dako	1:3200	Overnight at 4°C	Heat-induced (pH 6.0) by autoclave	EnVision
C4b	C4b	Rabbit	Abcam, Cambridge, United Kingdom	1:1600	Overnight at 4°C	Heat-induced (pH 6.0) by autoclave	EnVision
C5b-9, clone WU13-15	Neoepitope on C9 in the membrane attack complex	Mouse	Hycult Biotech, Plymouth meeting, USA	1:400	Overnight at 4°C	Heat-induced (pH 6.0) by autoclave	EnVision

IHC for pTau, amyloid-beta, GFAP, and Iba1 was performed using the Ventana BenchMark ULTRA staining system (Roche, Basel, Switzerland). Tissue sections were mounted on TOMO adhesive glass slides (Matsunami, Osaka, Japan) and deparaffinized. After blocking for endogenous peroxidase, antigen retrieval was performed by heating sections at 100 °C in Cell Conditioning 1 solution (pH 8.5) (Roche) for different durations per antibody (see Table 3). For detection of primary antibodies with 3,3′-diaminobenzidine tetrahydrochloride (DAB), Optiview DAB IHC detection kit (Roche) was used. Finally, the sections were mounted with Coverslipping film (Sakura Tissue-Tek, Leiden, The Netherlands).

IHC for CD68, HLA-DP/DQ/DR, C1q, C3d, C4b, and C5b-9 was performed manually. The sections were mounted on SuperFrost Plus glass slides (Menzel-Gläser, Braunschweig, Germany) and deparaffinized. Subsequently, the sections were blocked for endogenous peroxidase using 0.3% hydrogen peroxide in phosphate buffer saline (PBS; pH 7.4). The sections were immersed in sodium citrate buffer (10 mM sodium citrate, 5 M NaOH, dH_2_O, pH 6.0) and heated to 120 °C in an autoclave for antigen retrieval. Primary antibodies were diluted in normal antibody diluent (ImmunoLogic, Duiven, The Netherlands) and incubated overnight at 4 °C. Primary antibodies were detected using EnVision (Dako, Glostrup, Denmark). Between steps, the sections were washed in PBS. Subsequently, antibodies were visualized with DAB (Dako). After counterstaining with hematoxylin, the sections were mounted with Entellan (Merck, Darmstadt, Germany).

### Image analyses and quantitative assessment of immunostainings

For quantitative assessment, 2 regions of interest (ROI) were randomly selected within non-curved areas of each section containing all 6 cortical layers [28]. Within each ROI, contiguous microscopic fields arranged in columns perpendicular to the cortical surface of the cortex were photographed. Total surface, depending on the width of the cortex, could vary for each ROI and contained at least 2 columns. Images were taken using a × 10 objective on an Olympus BX 41 photomicroscope with a Leica MC 170 HD digital camera. The presence of DAB staining was quantified with ImageJ (NIH) using the color threshold plugin. Our outcome measurement was the percentage of DAB-stained area per marker, also referred to as immunoreactivity. In addition to immunoreactivity, we quantified the number of amyloid-beta and C4b plaques. For the amyloid-beta plaques, diffuse deposits were not taken into account and only dense plaques were quantified, defined as particles with an immunoreactive surface area of 100 μm^2^ or more [29, 30]. C4b-positive deposits of the same surface area were quantified as a measurement of the atypical appearing plaques as described in the “Results” section.

### Fluorescent triple stainings

Co-localization of C4b and CD68 or HLA-DP/DQ/DR with amyloid-beta and thioflavine S was visualized in the parietal section of 4 atypical AD cases and the temporal section of 2 typical AD cases. The typical AD cases served as positive controls and reference since localization of complement and microglia in classical-cored plaques is widely described in literature (for CD68 [20]/for complement [16, 19]).

After deparaffinization, the sections were submerged in sodium citrate buffer and heated to 120 °C in an autoclave. Subsequently, the sections were incubated using different combinations of primary antibodies: mouse IgG2a-anti-amyloid-beta (1:200), rabbit-anti-C4b (1:200), and mouse IgG1-anti-HLA-DP/DQ/DR (1:25) or mouse IgG1-anti-CD68 (1:300). Antibodies were diluted in normal antibody diluent (ImmunoLogic) and incubated overnight at 4 °C. Subsequently, the sections were incubated with the following secondary antibodies: goat-anti-mouse IgG2a Alexa Fluor dye 594, goat-anti-mouse IgG1 Alexa Fluor dye 647, and donkey-anti-rabbit Alexa Fluor dye 647 (1:250 dilution, ThermoFisher, Waltham, USA). For visualization of amyloid structures, the sections were counterstained with thioflavine S (1% in dH_2_O) and subsequently rinsed in 70% ethanol. Autofluorescence was blocked with 0.1% Sudan black in 70% ethanol for 5 min. Between steps, the sections were rinsed with PBS. Finally, the sections were enclosed with 80% glycerol/20% tris buffered saline. Representative pictures were taken with a Leica DMi8 inverted fluorescent microscope equipped with a Leica DFC300 G camera.

### Statistical analysis

Demographics of the typical and atypical AD groups were compared using Fisher’s exact test for categorical and Mann-Whitney *U* test for numerical and not normally distributed data. Outcome measures were compared between the 2 AD groups by using linear mixed model analysis. Linear mixed model analysis was used to adjust for the nested observations within cases. In the linear mixed model analyses, the group variable (typical versus atypical AD), the region (temporal versus parietal), and the interaction between group and region were added. Correcting for age and sex made the model less stable and was therefore not performed. An assumption to apply a linear mixed model is that residuals of outcome measurements are normally distributed. To meet this assumption, all outcome variables (pTau, amyloid-beta, GFAP, Iba1, HLA-DP/DQ/DR, CD68, C1q, C3d, C4b, number of amyloid-beta plaques, and number of C4b plaques) were transformed by taking the natural log of the (variable + 1). The covariance structure was set to unstructured. Using the linear mixed model, we answered if the difference in outcome measurement over the 2 regions was different between the 2 AD phenotypes (region × phenotype), also referred to as interaction effect. Both phenotypes showed a similar distribution over the 2 regions if no interaction effect was found. Statistical analysis was performed in IBM SPSS statistics version 22.0 (IBM SPSS Statistics, Armonk, NY, USA). Bonferroni correction was used to correct for multiple testing. Since we tested 11 outcome measurements (amyloid-beta, pTau, GFAP, Iba1, CD68, HLA-DP/DQ/DR, C1q, C3d, C4b, # amyloid-beta plaques, and # C4b plaques), statistical significance was set at *p* < .0045 (*p* < .05/11 outcome measurements) for each effect (region × phenotype and region) of the linear mixed model. Statistical significance was set at *p* < .05 for comparison of baseline characteristics.

## Results

### Atypical AD cases are younger than typical AD cases

The post-mortem cohort used for IHC consisted of 9 atypical AD cases and 10 typical AD cases. For a summary of initial clinical symptoms at presentation for each case, see Table 2. Most atypical AD cases presented with symptoms of aphasia, consisting of word-finding difficulties and spelling mistakes, combined with apraxia. None of our atypical cases retrospectively met the criteria for an isolated primary progressive aphasia [5]. One case presented with Parkinsonism and an alien hand syndrome, fitting a corticobasal syndrome during life. All 10 typical AD cases presented with memory complaints as most prominent initial symptom. Demographic characteristics of both AD phenotypes used for immunohistochemical analysis are shown in Table 4. Similar to the large cohort of 296 AD subjects (see Fig. 1), atypical AD patients selected for IHC analysis were younger at age of death and more often male. The disease duration, brain weight, post-mortem interval, disease severity, and ApoE genotype did not differ between groups.

**Table 4:** Demographic characteristics of the AD cases used for immunohistochemical analysis

	Typical AD (*n* = 10)	Atypical AD (*n* = 9)	*p*-value
Male, *n*	2	7	< .05
Age at death	81 (± 10)	63 (± 3)	< .01
Disease duration	5 (± 3)	4 (± 2)	.78
Brain weight (grams)	1043 (± 146)	1117 (± 161)	.32
PMI (h:min)	6:19 (± 1:48)	5:51 (± 1:33)	.66
NFT stage [7] *n* per stage 4/5/6	2/5/3	0/4/5	.46
Amyloid stage [7] *n* per stage O/A/B/C	0/0/10	0/1/8	.47
ApoE genotype *n* per category 32/33/42/43/44	1/2/0/6/1	0/3/1/3/2	.48

Data in mean (± SD). Age at death and disease duration in years. Mann-Whitney *U* test for continuous data. Fisher’s exact test for categorical data

*AD* Alzheimer’s disease, *NFT* neurofibrillary tangle, *PMI* post-mortem interval

### Distribution of pTau and amyloid-beta in typical and atypical AD

**Fig. 2:**
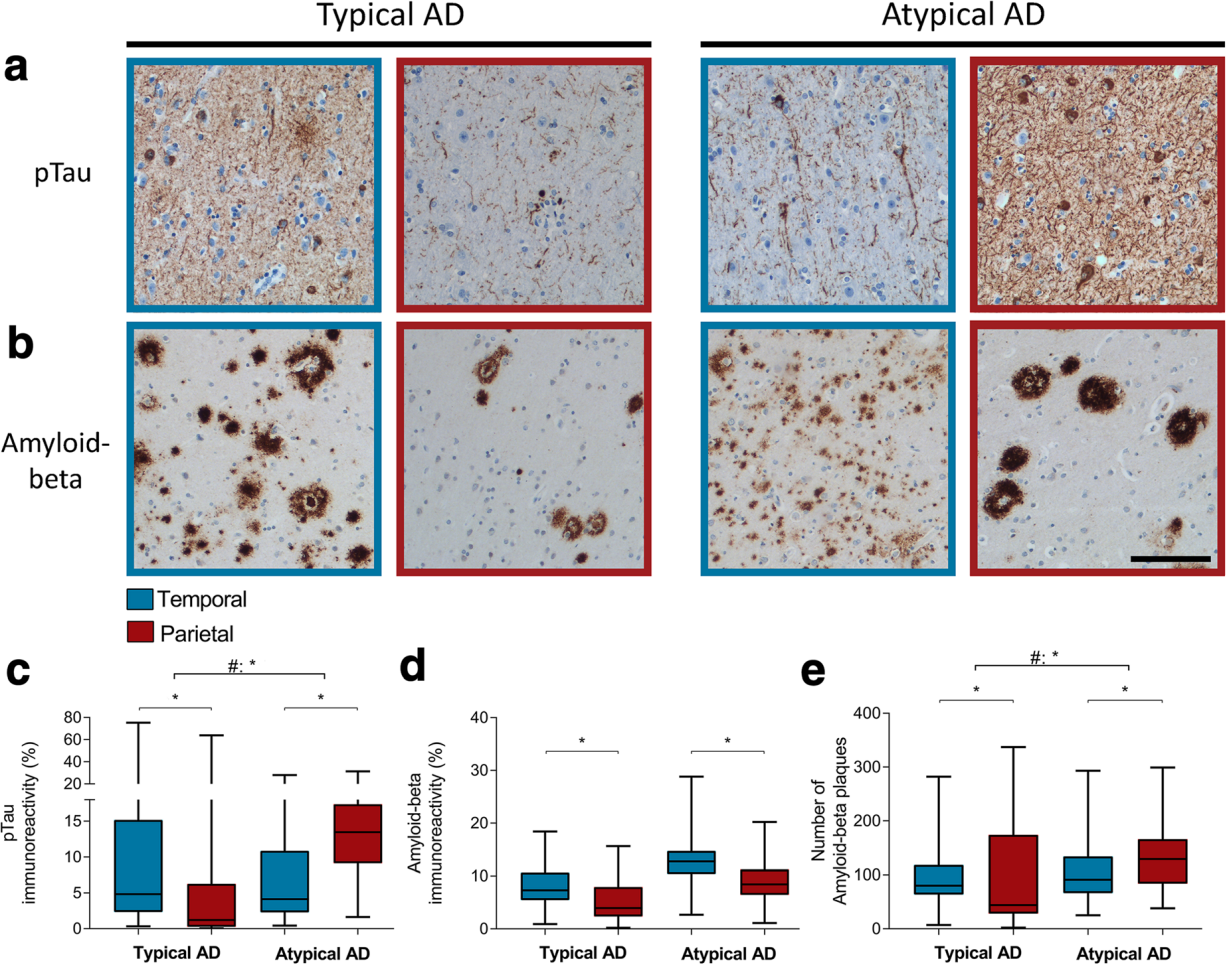
pTau and amyloid-beta distribution in typical AD and atypical AD. **a** In typical AD, the temporal cortex (blue border) shows more immunoreactivity for pTau than the parietal cortex (burgundy border). This distribution is inversed in atypical AD (boxplot in **c**). **b** Although both typical and atypical AD show more overall amyloid-beta immunoreactivity in the temporal cortex compared to the parietal region, the atypical AD group shows increased number of amyloid-beta plaques in the parietal compared to temporal section (boxplot in **e**). Bar represents 100 μm. **c**, **d**, and **e** Boxplots showing pTau immunoreactive area (%), amyloid-beta immunoreactive area (%), and the number of amyloid-beta plaques, respectively, in the temporal and parietal section of both AD phenotypes. Data shown as median (bar), 1st and 3rd quartile (box boundaries), and min to max (error bars). A difference in distribution over the two regions between the two AD phenotypes is indicated by # (Table 5), * *p* < .0045

Immunohistochemistry for pTau showed neuronal inclusions as well as neuritic threads (Fig. 2a). Typical AD cases showed more pTau immunoreactivity in the temporal compared to the parietal section (Fig. 2c). This was contrary to the pTau distribution in atypical AD cases, in which the parietal section showed more immunoreactivity compared to the temporal section. In addition, the distribution of pTau over the 2 regions differed significantly between the 2 phenotypes (Table 5).

**Table 5:** Results for the linear mixed model of transformed immunohistochemistry variables

Transformed variable	Fixed Effect	Phenotype	Results for linear mixed model	
			Beta-coefficient	95% CI
pTau	Region x phenotype		1.53*	[1.33 – 1.72]
	Region	Typical AD	- 0.82*	[- 0.95 – - 0.68]
		Atypical AD	0.71*	[0.57 – 0.85]
Amyloid-beta	Region x phenotype		0.04	[- 0.06 – 0.15]
	Region	Both	- 0.46*	[- 0.54 – - 0.39]
GFAP	Region x phenotype		1.25*	[1.02 – 1.48]
	Region	Typical AD	- 1.71*	[- 1.87 – - 1.56]
		Atypical AD	- 0.46*	[- 0.63 – - 0.29]
Iba1	Region x phenotype		0.10	[0.002 – 0.19]
	Region	Both	- 0.47*	[- 0.53 – - 0.41]
CD68	Region x phenotype		- 0.43*	[0.38 – 0.48]
	Region	Typical AD	- 0.13*	[- 0.17 – - 0.10]
		Atypical AD	0.29*	[0.26 – 0.33]
HLA-DP/DQ/DR	Region x phenotype		0.97*	[0.88 – 1.06]
	Region	Typical AD	- 0.08*	[- 0.14 – - 0.03]
		Atypical AD	0.89*	[0.82 – 0.96]
C1q	Region x phenotype		- 0.05	[- 0.15 – 0.06]
	Region	Both	0.03	[- 0.05 – 0.10]
C3d	Region x phenotype		0.74*	[0.62 – 0.86]
	Region	Typical AD	- 0.39*	[ - 0.47 – - 0.31]
		Atypical AD	0.36*	[0.26 – 0.45]
C4b	Region x phenotype		0.95*	[0.84 – 1.07]
	Region	Typical AD	- 0.24*	[- 0.32 – - 0.17]
		Atypical AD	0.71*	[0.62 – 0.80]
# of amyloid-beta plaques	Region x phenotype		0.58*	[0.41 – 0.75]
	Region	Typical AD	- 0.33*	[- 0.44 – - 0.21]
		Atypical AD	0.25*	[0.13 – 0.37]
# of C4b plaques	Region x phenotype		1.84*	[1.58 – 2.11]
	Region	Typical AD	- 0.39*	[- 0.56 – - 0.22]
		Atypical AD	1.46*	[1.25 – 1.66]

Results of the linear mixed model for analyzed immunohistochemistry variables are shown. All variables were transformed: ln(variable + 1). We tested if the distribution over the 2 regions was different between the 2 AD phenotypes, defined as the interaction effect: region × phenotype. When an interaction effect was found, the beta-coefficient is shown per phenotype. To correct for multiple testing, a *p* value < .0045 was considered significant (*p* < .05/11 outcome measurements) and indicated with *

*AD* Alzheimer’s disease, *CI* confidence interval

*#* Number

Amyloid-beta immunoreactivity was present in the form of diffuse deposits, dense plaques, and classical cored plaques (Fig. 2b). Both the typical and atypical AD group showed more immunoreactivity for amyloid-beta in the temporal than parietal section (Fig. 2d), and no difference in distribution was observed (Table 5). While total immunoreactivity levels did not show differences between the 2 phenotypes, the number of dense plaques with an immunoreactive surface area of 100 μm^2^ or more did show a significant difference in distribution over the 2 regions between the 2 phenotypes (Fig. 2e) (Table 5). In contrast to the typical AD group, more dense plaques were observed in the parietal section of the atypical AD group. Besides a difference in plaque number, we observed a contrast in plaque morphology between the two groups, which will be addressed below.

### Glial activation is increased in atypical AD

**Fig. 3:**
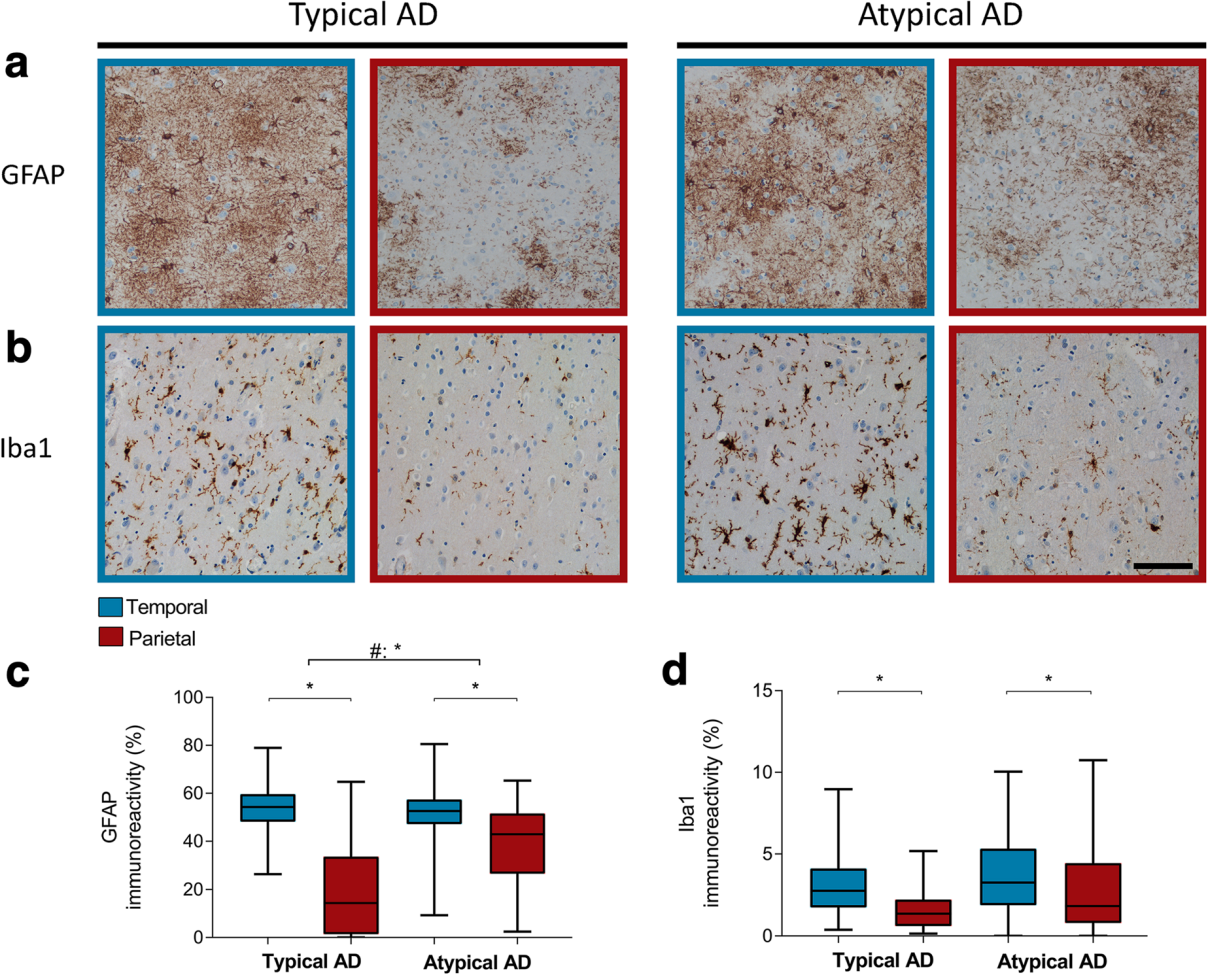
GFAP and Iba1 immunoreactivity is temporal dominant in both AD phenotypes. **a**, **b** The temporal cortex (blue border) shows more GFAP and Iba1 immunoreactivity respectively than the parietal cortex (burgundy border) in typical and atypical AD. Bar represents 100 μm. **c**, **d** Boxplot showing GFAP and Iba1 immunoreactive area (%) in the temporal and parietal section, respectively, of both AD phenotypes. Atypical AD shows relatively more GFAP immunoreactivity in the parietal cortex than typical AD (Table 5). Iba1 distribution is not different between the two phenotypes. Data shown as median (bar), 1st and 3rd quartile (box boundaries), and min to max (error bars). *p < .0045

Staining for GFAP-positive astrocytes showed variably sized star-like GFAP-positive structures in all AD cases (Fig. 3a). Both phenotypes showed higher levels of immunoreactivity for GFAP in the temporal section compared to the parietal section. Atypical AD cases showed relatively more GFAP immunoreactivity in the parietal cortex compared to typical AD cases (Fig. 3c; Table 5).

Iba1 immunostaining showed positivity in both the cell soma as well as the processes of the microglia, mostly in the form of ramified microglia (Fig. 3b). Both AD groups showed a similar temporal dominancy for Iba1.

**Fig. 4:**
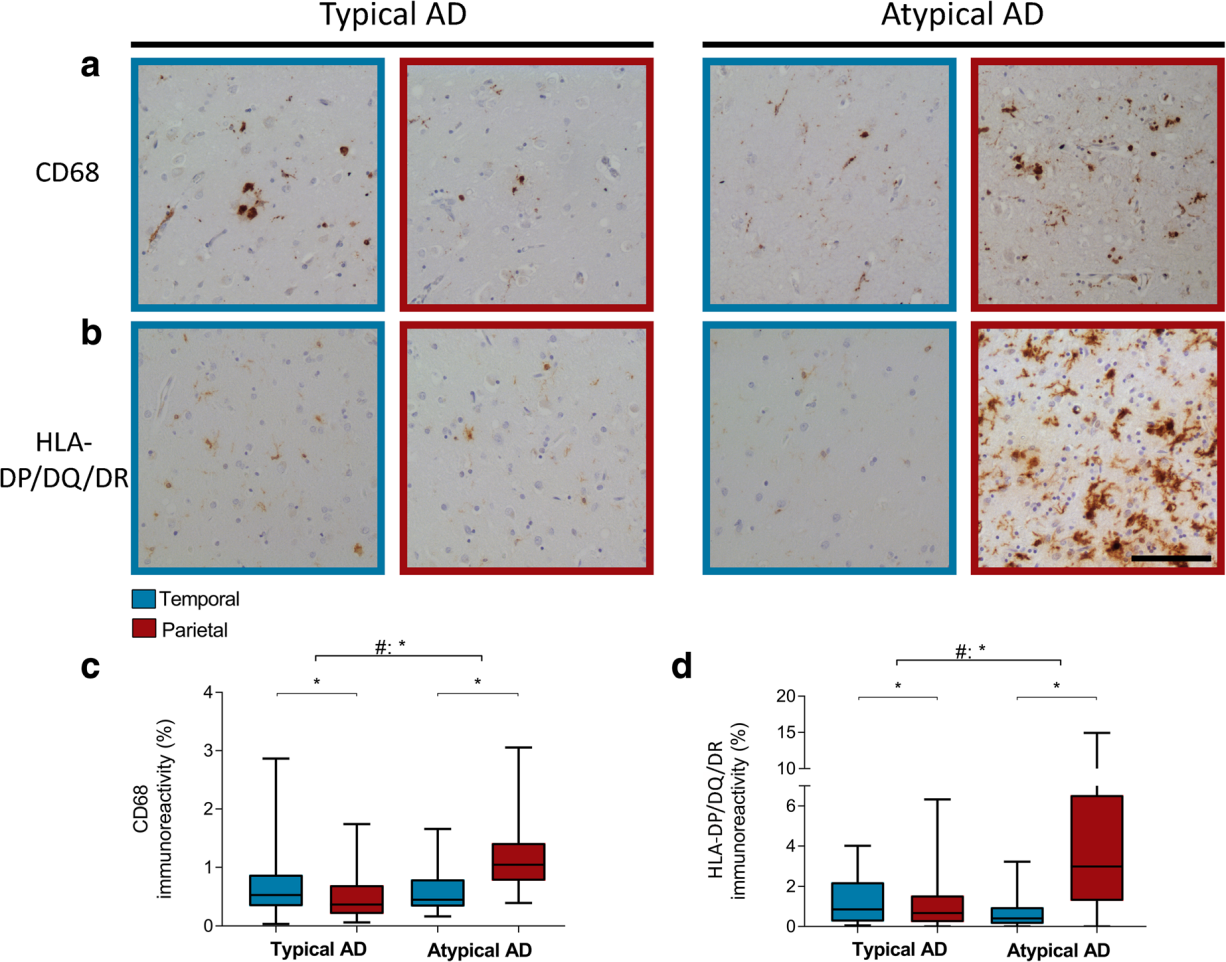
CD68 and HLA-DP/DQ/DR immunoreactivity show a parietal dominant distribution in atypical AD. **a**, **b** In typical AD, the temporal section (blue border) shows more CD68 and HLA-DP/DQ/DR immunoreactivity than the parietal section (burgundy border). In contrast, atypical AD shows more CD68 and HLA-DP/DQ/DR immunoreactivity in the parietal than the temporal section. Note the relative difference in immunoreactivity in the parietal section of atypical AD compared to the temporal section in typical AD. Bar represents 100 μm. **c**, **d** Boxplot showing CD68 and HLA-DP/DQ/DR immunoreactive area (%) in the temporal and parietal section, respectively, of both AD phenotypes. Linear mixed model shows a different distribution over the 2 regions between phenotypes (#) (Table 5). *p < .0045

Activated microglia were stained using CD68 and HLA-DP/DQ/DR. Whereas CD68 positivity was mostly found in the soma of microglia, HLA-DP/DQ/DR showed a prominent staining in the processes of microglia (Fig. 4a, b).

Both markers showed a different distribution over the 2 regions between the 2 AD phenotypes (Fig. 4c, d; Table 5). Atypical AD cases showed more immunoreactivity for CD68 and HLA-DP/DQ/DR in the parietal compared to the temporal section, which was in contrast to the typical AD cases. In addition, the levels of CD68 and HLA-DP/DQ/DR immunoreactivity were relatively high in the parietal section of atypical AD compared to the temporal section of typical AD.

### Increased presence of complement proteins in atypical AD

To visualize different parts of the complement cascade, we stained for C1q, C3d, and C4b, representing the start of the cascade, as well as for C5b-9, defining the end-stage of the cascade and forming the membrane attack complex.

**Fig. 5:**
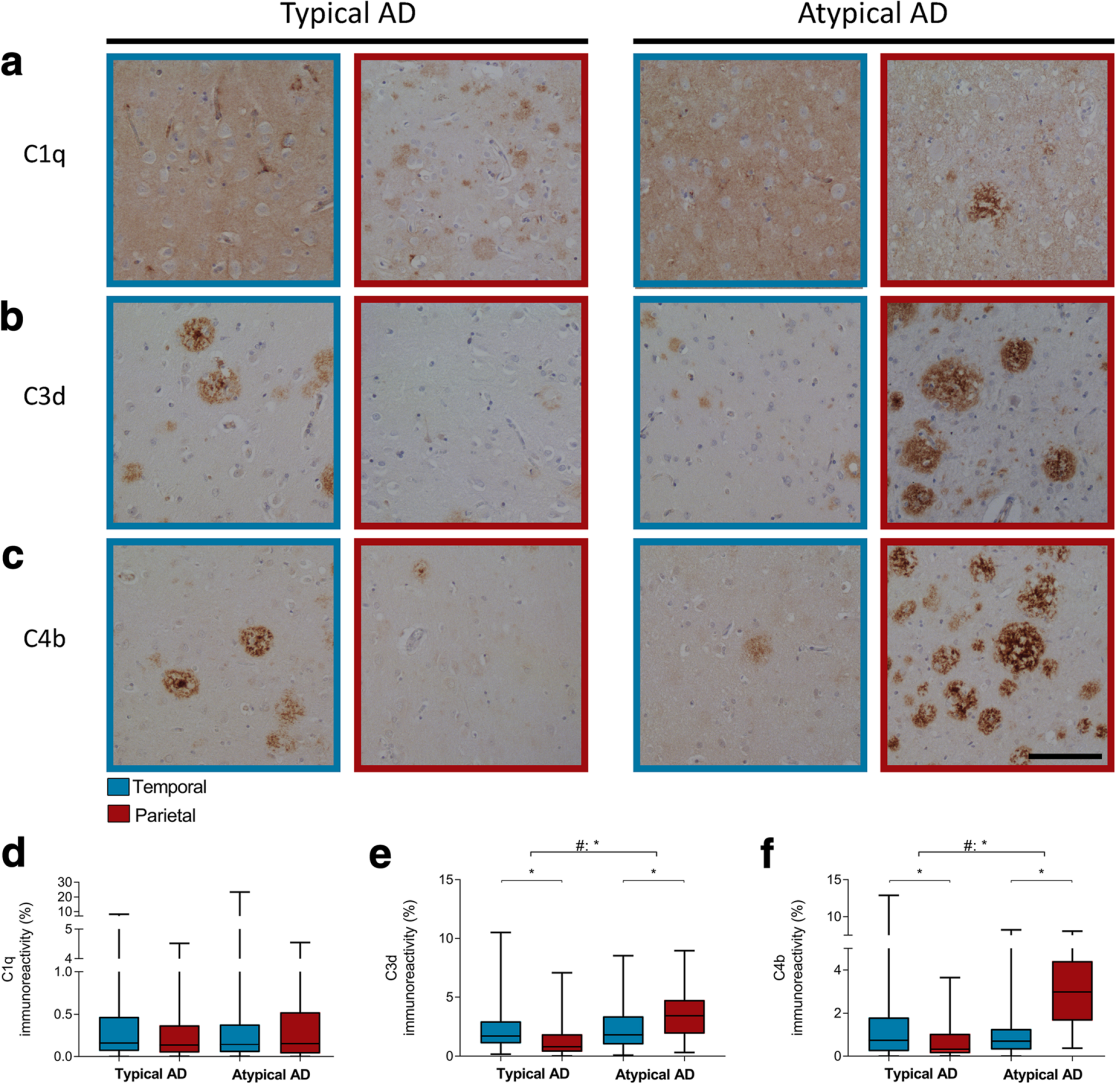
Distribution of C3d and C4b differs between AD phenotypes. **a** In both typical and atypical AD, C1q deposition in the temporal cortex (blue border) is more diffuse than in the parietal cortex (burgundy border). **b**, **c** In typical AD, the temporal cortex shows more immunoreactivity for C3d and C4b than the parietal cortex. This distribution is inverted in atypical AD, showing more immunoreactivity for C3d and C4b in the parietal than temporal section. Bar represents 100 μm. **d**, **e**, and **f** Boxplots of immunoreactive area (%) for C1q, C3d, and C4b, respectively, in the temporal and parietal section of both AD phenotypes. Linear mixed model shows a different distribution for C3d and C4b over the 2 regions between phenotypes (#) (Table 5). *p < .0045

C1q immunoreactivity was observed as a weak diffuse staining in the form of plaque-like structures, as punctuate staining of the neuropil, and sometimes in neurons (Fig. 5a). While the plaque-like structures were more often observed in the parietal section, the punctate staining of the neuropil was more prominent in the temporal section of both AD groups. No difference in C1q immunoreactivity was observed between regions or AD phenotypes (Fig. 5d; Table 5).

Compared to C1q, staining for C3d and C4b showed an intense plaque-like staining, which had a morphology resembling that of compact and classical cored plaques (Fig. 5b, c). Levels of immunoreactivity for C3d and C4b in typical AD were higher in the temporal compared to the parietal cortex (Fig. 5e, f; Table 5). In contrast, atypical AD showed higher immunoreactivity levels for both markers in the parietal section compared to the temporal section.

Analysis of C5b-9 exposed very low to no immunoreactivity in both regions of both phenotypes (data not shown). For this reason, no quantification or statistical analysis was performed for C5b-9. The few structures that were C5b-9 positive included parenchymal and meningeal vessels. The serum within these vessels also stained positive for C5b-9.

Our data show a different distribution in temporal and parietal regions between both AD phenotypes for complement factors C3d and C4b. Both complement factors were mostly abundant in the parietal section of atypical AD.

### Different plaque appearance in typical and atypical AD

**Fig. 6:**
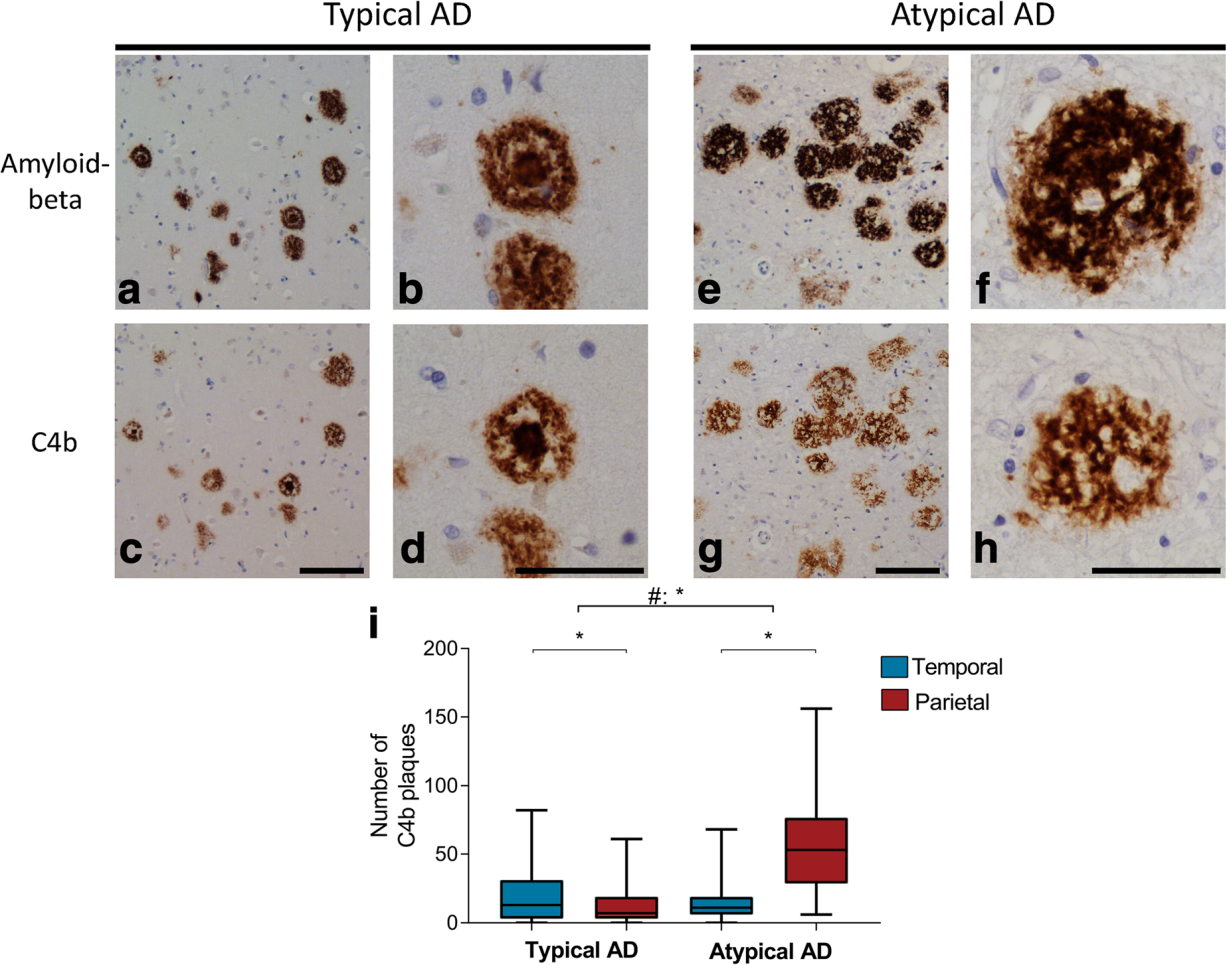
Plaques in atypical AD show a different morphology compared to plaques in typical AD. **a**–**d** In typical AD cases, the morphology of amyloid-beta (**a** for overview, **b** for detail) and C4b (**c** for overview, **d** for detail) deposits come in the form of diffuse, dense, or classical cored plaques. **e**–**h** In atypical AD cases, amyloid-beta (**e** for overview, **f** for detail) and C4b (**g** for overview, **h** for detail) deposits show a distinct morphology, being coarse-grained and affecting a larger surface area (> 100 μm^2^) compared to classical cored plaques. Pictures are taken in the region with the highest number of amyloid-beta plaques, being temporal for typical AD and parietal for atypical AD (Fig. 2e). Bars represent 100 μm. **i** Boxplot of number of coarse-grained plaques quantified using C4b staining in the temporal and parietal cortex of typical and atypical AD. Linear mixed model shows a different distribution for coarse-grained plaques over the 2 regions between phenotypes (#) (Table 5). This distribution is parietally dominant in atypical AD. *p < .0045

**Fig. 7:**
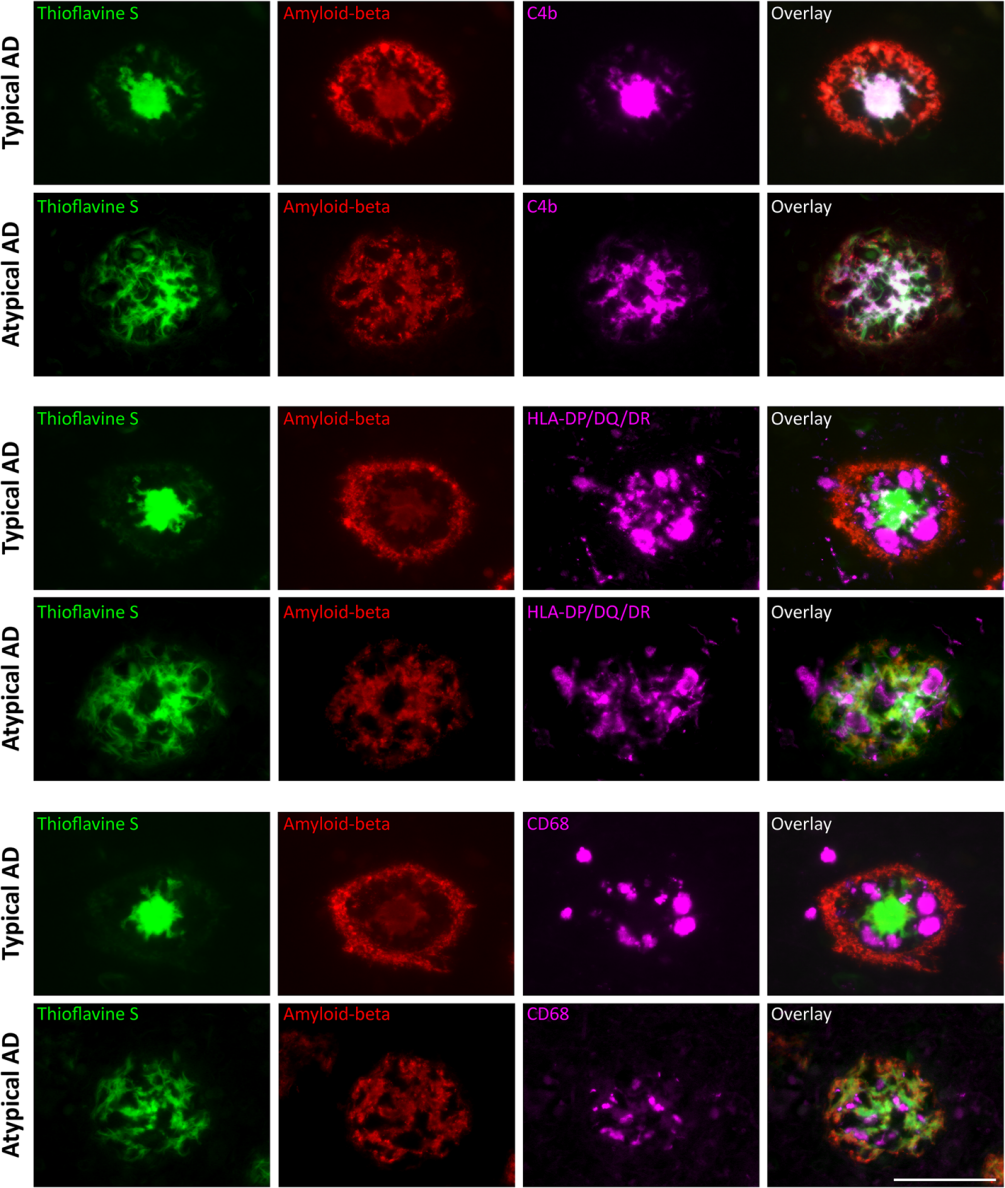
Different plaque morphology in the parietal cortex of atypical AD. First row: in typical AD, classical cored plaques in the temporal cortex show an organized staining pattern with a corona showing merely amyloid-beta positivity versus a core positive for thioflavine S, amyloid-beta, and C4b. Second row: in atypical AD, fibrillar plaques in the parietal cortex show co-localization of thioflavine S, amyloid-beta, and C4b in the form of fibrils throughout the whole plaque. Third row: in typical AD, CD68-positive microglia are localized between the core and corona of classical cored plaques. Fourth row: in atypical AD, CD68-positive microglia localization is less organized. Fifth + sixth row: this different distribution within plaques between the two phenotypes also holds for HLA-DP/DQ/DR-positive microglia. Bar is applicable to all images and represents 100 μm

Looking at the morphology of plaques stained by amyloid-beta and C4b, we observed a difference in appearance between the 2 AD phenotypes. Amyloid-beta and C4b immunoreactive plaques in the parietal section of atypical AD cases showed a more granular composition compared to deposits in the temporal section of this phenotype or compared to deposits in both regions of the typical AD cases (Fig. 6). The surface area positive for C4b of these coarse-grained plaques was larger (> 100 μm^2^) than that of typical plaques. Atypical AD cases had more of these C4b plaques in the parietal compared to the temporal cortex, which was contrary to typical AD cases (Fig. 6i; Table 5). The coarse-grained plaques observed in the parietal cortex of atypical AD triple-stained for C4b, amyloid-beta, and thioflavine S (Fig. 7). This staining pattern was compared with that of classical-cored plaques observed in typical AD. In classical-cored plaques, C4b, amyloid-beta, and thioflavine S co-localized in the core of the plaque, while the corona only stained for amyloid-beta. Compared to cored plaques, coarse-grained plaques showed a fibrillar, less organized morphology with co-localization of C4b, amyloid-beta, and thioflavine S all over the plaque surface. Since an increased presence of activated microglia in atypical AD was observed, the localization of CD68 and HLA-DP/DQ/DR with cored and coarse-grained plaques was compared (Fig. 7). Like cored plaques, coarse-grained plaques were associated with clusters of CD68 and HLA-DP/DQ/DR-positive microglia. In cored plaques, activated microglia were located between the core and corona of the plaque. In coarse-grained plaques, the localization of CD68 and HLA-DP/DQ/DR-positive microglia was less structured and positive microglia appeared throughout the plaque. This data supports a morphological difference between cored and coarse-grained plaques, of which the latter occurs prominently in atypical AD (Fig. 6i).

## Discussion

Atypical AD is characterized by a different distribution of NFTs when compared with typical AD. As expected, we observed that also the occurrence of pTau is differently distributed in typical and atypical AD. Here, we show for the first time that the distribution of both activated microglia and complement factors between the temporal and parietal lobe differentiates atypical from typical AD cases. In addition, atypical AD cases are characterized by the presence of plaques with an abnormal morphology, highlighted by an alternative localization of microglia and presence of complement proteins.

In this study, typical and atypical AD were defined according to the distribution of NFTs as well as their clinical presentation. In our cohort, atypical AD cases were younger and more often male than typical AD cases. This was also seen in an earlier study by Murray and colleagues [31], indicating that an atypical distribution of pathology seems to be more common in men and at a younger age. In line with this, another study showed that cases with EOAD showed higher mean levels of NFTs in the parietal lobe, when comparing EOAD to LOAD, irrespective of clinical presentation [32]. When taking symptomology into account, this parietal dominant tau distribution is especially common in the PCA variant of atypical AD [11]. Therefore, the current atypical AD cohort defined by parietal dominant NFTs represents only a subgroup of atypical AD. Results from this study do not necessarily apply for patients with a logopenic or behavioral phenotype, as tau pathology seems to be differently distributed in those cases [11, 33]. Besides neuropathological studies, also clinical studies report that an atypical clinical presentation is more common at a younger age [3]. Regarding differences in gender, an atypical presentation is not per se more common in men [3]. However, AD presenting at late-onset is more common in women [34], explaining the relative high number of female subjects in typical AD cohorts.

We did not observe a different distribution of total amyloid-beta immunoreactivity between 2 AD phenotypes. This is in line with other studies reporting that amyloid-beta distribution is not different between AD subtypes [9, 32, 35–37]. However, when quantifying for number of dense amyloid-beta plaques, atypical AD cases showed a parietal dominant distribution compared to typical AD cases, which showed a temporal dominant distribution. This distinction in number of plaques was also reported by Hoff and colleagues who compared cases with PCA to typical AD cases using stereology [38]. These results indicate that although the distribution of overall amyloid-beta immunoreactivity is similar, the number of dense amyloid-beta plaques may differ between AD phenotypes. In addition to the number of dense amyloid-beta plaques, structural differences in amyloid-beta plaques might be associated with the pathological and clinical differences between typical and atypical AD.

In the present study, we observed a clear morphological difference between classical-cored plaques in typical AD and dense plaques in atypical AD cases. Dense plaques in atypical AD cases have a coarse-grained structure, as observed with amyloid-beta immunostaining and thioflavine S staining. In addition, these coarse-grained plaques showed a strong immunoreactivity for complement. Multiple studies have reported complement proteins to be associated with amyloid-beta deposits [19, 39]. However, a difference in complement activation between AD subtypes has so far not been described. The increased presence of complement in coarse-grained plaques in atypical AD cases supports a difference in amyloid structure between typical and atypical AD. Most likely, the amyloid structure of fibrillar plaques favors a strong binding and activation of complement factors, which in turn could act as opsonins for phagocytosis carried out by microglia [17, 40, 41]. A structural variation in amyloid-beta fibrils in combination with a difference in binding of amyloid associated proteins may underlie the observed difference in the occurrence of neuroinflammation, pTau, and NFTs between typical and atypical AD. The relation between amyloid-beta, complement, and microglia is underlined by a study in APP transgenic mice deficient for C3 showing less cognitive problems and more amyloid-beta plaques compared to APP mice not deficient for C3 [42]. The amyloid-beta plaques in the C3 knockout mice showed less microglial co-localization. Together, these findings suggest that structural differences in amyloid deposits in atypical AD may directly be related to complement and microglial activation.

The distribution of NFT and pTau pathology is clearly associated with the presence of activated microglia in AD variants. Recent animal studies have shown that microglia are capable of both internalizing [43] and excreting pTau [24, 44], suggesting that microglia contribute to the spreading of the pathology. Indeed, when mice are depleted for microglia, spreading of tau pathology is significantly reduced [23, 24]. In addition, activated microglia could also contribute or induce tau hyperphosphorylation in neurons [45]. These studies indicate that microglial activation drives the spreading of pathology and stimulates neurofibrillary degeneration. Interestingly, recent evidence from a pathological study suggests that activation of microglia may precede tau pathology in chronic traumatic encephalopathy [46], which implicates that tau pathology may be a consequence rather than a cause for microglial activation.

The aforementioned demographical differences in age and sex may influence the neuroinflammatory response. Former studies have shown various results on the correlation between sex, age, and microglial activation in both humans and animal models. Schwarz et al. showed that during early development, male rats have more microglia within the parietal cortex compared to female rats. However, during juvenile and early adulthood, this balance switches, indicating that sex hormones influence microglial colonization at different time points in rats [47]. In human studies, contradictory results are published for the effect of age. In healthy controls, aging is correlated with a more primed microglial state. Nevertheless, this was shown to be different in diseased cases, in which increasing age is associated with a diminished neuroinflammatory response [25]. These studies suggest that sex and age influence microglia activation. However, whether differences in sex and age contribute to differences in regional distribution, as observed in the current study between AD subtypes, remain elusive.

## Conclusions

The results of this study are in line with the assumption that the fibrillar structure and protein composition of different plaques may be relevant for the difference in regional vulnerability among AD phenotypes. In addition, our results support a role for activated microglia and complement factors in the atypical spreading of pathology in AD subtypes. In this study, we focused at 2 brain regions in a subset of atypical AD. It would be interesting to expand this study on the role of neuroinflammation to various brain regions in a larger atypical AD cohort. More clinicopathological studies of AD variants (e.g., logopenic and behavioral frontal) are needed to better understand the relation between amyloid-beta, pTau, and related pathological mechanisms. Future research should focus on how variable disease mechanisms underlie the regional susceptibility in different brain regions leading to different clinical AD subtypes.

## Abbreviations

AD: Alzheimer’s disease
DAB: 3,3′-Diaminobenzidine tetrahydrochloride
EOAD: Early-onset Alzheimer’s disease (< 65 years)
FFPE: Formalin-fixed paraffin-embedded
GFAP: Glial fibrillary acidic protein
IHC: Immunohistochemistry
IQR: Inter-quartile range
LOAD: Late-onset Alzheimer’s disease (≥ 65 years)
NBB: Netherlands Brain Bank
NFT: Neurofibrillary tangle
PBS: Phosphate buffer saline
PCA: Posterior cortical atrophy
PMI: Post-mortem interval
pTau: Phosphorylated Tau
ROI: Region of interest
